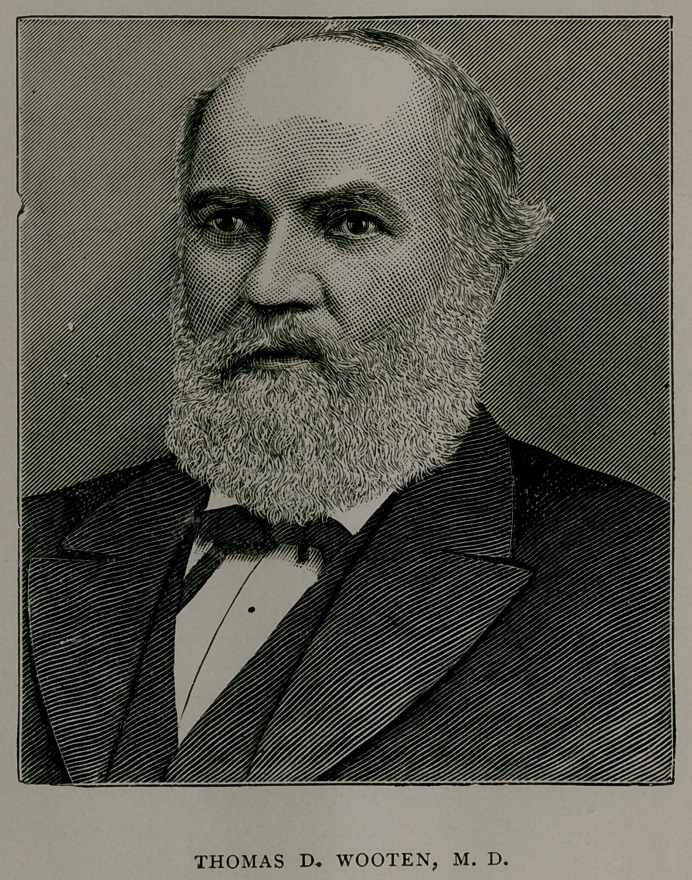# Thomas Dudley Wooten, M. D., President Austin Dist. Med. Soc., Etc.

**Published:** 1887-11

**Authors:** 


### BIOGRAPHY.

### THOMAS DUDLEY WOOTEN, M. D.

Thomas Dudley Wooten, now of Austin, was born in Barren County, Kentucky, on the 6th of March, 1829. His parents were from Virginia, having removed to Kentucky in the early settlement of its southern portion. Acquiring extensive landed interests in the new country, his father, Joseph Wooten, by that thrift and attention to administrative details so characteristic of the better class of the old southern farmers, rapidly established a large plantation, with all the appointments of field, shop, mill and stock farm which rendered the early homesteads of Kentucky and Tennessee so attractive, so efficient and so valuable as training schools of industry and self-reliance to the youths who shared their labor and management.

The subject of this sketch was the youngest but one of several sons, and the death of his father left him, at the age of fifteen years, virtual master of the farm and slaves, with all the toil and responsibility incident to the successful control and administration of the large estate. These he assumed and discharged for several years, receiving and acquiring in the meanwhile such education as the schools of the country permitted and his own diligent reading and study, prosecuted at night and in the intervals of labor, afforded.

Nearing his majority he began the study of medicine, and after a year's reading in the office of Dr. George Rogers in the town of Glasgow, he entered the Medical Department of the University of Louisville, in the fall of 1851. That venerable institution was then second to none in the Union in its medical faculty and course of instruction, numbering among its professors such men as the elders Flint, Gross and Yandell, Drake, Caldwell, Silliman and Miller. Before completing his medical course he was married to Miss. Henrietta C. Goodall, the daughter of Dr. Turner Goodall, a successful practitioner of Tompkinsville, Monroe County, Kentucky.

Graduating in the spring of 1853, Dr. Wooten located at Tompkinsville and entered upon the active practice, with the usual experiences and successes of the young physician in a country town. In the early part of 1856 he removed to Springfield in southwestern Missouri, where for the first year he was engaged in building and improving a home and establishing a farm near the young and growing city. This accomplished, he at once resumed his professional life, and was soon established in a lucrative and constantly increasing practice. Although then, as now, he pursued the practice of his chosen science in all its branches, and set up no claims as a specialist, yet from the first his marked success and skill in surgery, gynaecology and treatment of diseases of the eye rendered his reputation in those special directions a matter of public and professional comment and approval. When the war began he had laid the foundations of a confortable fortune and a successful career, which were swept away by that great revolution.

Missouri's peculiar attitude toward the Rebellion is a matter of history. She occupied the position at first of neutrality, and afterwards of armed defence of her own soil and sovereignty against invasion by the Union armies. By the necessities of the case she naturally allied herself to the seceding States, and eventually the force of circumstances and the sympathies of her people compelled a merger of her military organization in that of the Southern Confederacy.

When the State decided to maintain her position of sovereign neutrality and called for troops, in June, 1861, Dr. Wooten enlisted as private in the company of Col. Richard Campbell. Upon the organization of the forces in southwestern Missouri, a little later, he was made surgeon of Foster's regiment, being the Second regiment, Seventh division, Missouri State Troops, commanded by Gen. McBride. After the battle of Oak Hills (Wilson's Creek), Aug. 10th, 1861, he was appointed Chief Surgeon of McBride's Division. Following the battle of Pea Ridge (Elk Horn), he was appointed Surgeon General of all the Missouri forces, *vice* Dr. Snodgrass resigned. When the Missouri army was turned over to the Confederacy and, together with the Arkansas troops, formed into the First Army Corps of the West, he was selected by the medical staff of the army as Medical Director of the Corps, with rank on the staff of Maj.-Gen. Sterling Price commanding.

Upon the transfer of this command to the east of the Mississippi river, and after the battle of Farmington, Gen. Price was placed in command of the District of Tennessee, embracing the States of Tennessee, Mississippi, Louisiana and part of Alabama, and Dr. Wooten was made Medical Director of the District. At that time the field and hospital service being consolidated and there being some fifteen thousand wounded and sick in hospital and repeated engagements in the field, the labor of the chief medical officer was immense and required the exercise of the greatest vigilance, firmness and skill, all of which were displayed abundantly and satisfactorily by Dr. Wooten. When Price was again ordered to the West and placed in command of the District of Arkansas, Dr. Wooten retained position on his staff and served as Medical Director of that district to the end of the war, being for a time on the staff of General Magruder, during Price's last raid into Missouri.

Dr. Wooten's rapid rise and sustained success in the army were somewhat remarkable. Only thirty-two years old at the outbreak of hostilities, with but four years residence and acquaintance in Missouri, with no previous military experience, no political prestige or professional affiliations, he enlisted as a private, and after a few months, in competition with some of the most eminent and influential medical men of St. Louis and the West, he rose to the highest medical rank in the service of the State, and to the Medical Directorship of the Western Army Corps, retaining to the close, his position on the staff, and his place in the confidence and affection of Missouri's devoted old warrior and chieftain.

At the end of the war, completely ruined in fortune, though influenced to locate in some of the larger cities, he settled at Paris, Texas, in 1865, where he soon built up a very large practice. Still maintaining his early aptitude and skill in the directions above referred to, and reinforced by a four years experience in the active and stirring emergencies of the field and hospital, his success in all the more difficult tasks of surgery and general practice fully sustained in civil life the reputation he had won in military circles. During the ten years he resided in Paris, besides a large local patronage, he drew his patients from a large part of northern and eastern Texas and from Arkansas, Louisiana and the Indian Territory.

Removing to Austin in January, 1876, he has continued the same assiduous devotion to his profession and been liberally rewarded with the same recognition of his professional skill at home and from a distance.

His reputation and success as a surgeon are part of the history of the medical profession of the State, and require no detailed mention. His has been a life rather of active attention to the duties of a laborious practice than an attempt to gain fame by devotion to the literary field of thought and investigation. He has never contributed much to medical literature, rarely writing for the reviews and seldom appearing before the State and local Associations except when officially called upon. Reports of his operations and notes of his cases would have formed valuable additions to current professional discussions, but he has apparently had neither the time nor the inclination to preserve or make them public. He has been a prominent member of the State Medical Association, and is a member of the American Medical Association and the American Public Health Association, to both of which bodies he has been a delegate from Texas. He was elected at the last regular meeting of the State Association a delegate to the International Medical Congress at Washington.

Upon the organization of the District Medical Society at Austin a few weeks since, he was elected its first President.

When the University of Texas was finally inaugurated, in 1881, Dr. Wooten was appointed by Gov. Roberts one of the original Regents of that institution, to which position he was re-appointed by Gov. Ireland. He has from the first been a most active and earnest friend of the University, and has labored for its successful and efficient establishment with a zeal and fidelity that have faltered under none of the discouraging indifference and hostility to the State's great seat of learning. Being the only member of the Regency resident at the capital, the greater part of the incessant vigilance and labor required to properly administer the affairs of the institution has fallen upon him.

In January, 1886, Dr. Ashbel Smith, President of the Board, having died the autumn previous, Dr. Wooten was unanimously elected President of the Board of Regents of the University of Texas, which position he still holds.

## Figures and Tables

**Figure f1:**